# Sodium-Glucose Co-transporter 2 Inhibitor-Associated Euglycemic Diabetic Ketoacidosis After Bariatric Surgery: A Case and Literature Review

**DOI:** 10.7759/cureus.17093

**Published:** 2021-08-11

**Authors:** Vaishali Kapila, Joel Topf

**Affiliations:** 1 Medicine, Central Michigan University College of Medicine, Mt. Pleasant, USA; 2 Nephrology, Ascension St. John Hospital, Detroit, USA; 3 Nephrology, Oakland University William Beaumont School of Medicine, Rochester, USA

**Keywords:** euglycemic diabetic ketoacidosis, dka, sglt2 inhibitor, hypokalemia, ketogenic diet

## Abstract

Sodium-glucose cotransporter-2 inhibitors (SGLT2i) reduce cardiovascular, kidney, and overall mortality. SGLT2i are also associated with a rare adverse event, euglycemic diabetic ketoacidosis (EDKA). This report describes a case of EDKA one day after bariatric surgery in a 51-year-old female with type 2 diabetes mellitus managed with the SGLT2i, canagliflozin. She was following a ketogenic diet for three weeks prior to surgery. The patient made a steady recovery with rapid anion gap closure followed by prolonged non-anion gap metabolic acidosis. Her medical record was tagged with a life-threatening reaction to SGLT2i. The risk of EDKA from SGLT2i may be increased by a low carbohydrate diet or postoperative status. Our case was complicated by hypokalemia, exemplifying the need for aggressive electrolyte management. Further guidance is needed to manage risk factors provoking EDKA and the use of SGLT2i therapy after an episode of EDKA.

## Introduction

Diabetic ketoacidosis is an acute complication of diabetes characterized by the triad: uncontrolled hyperglycemia (>250 mg/dl), metabolic acidosis (arterial pH <7.3 and serum bicarbonate <18 mEq/L), and ketosis. Euglycemic diabetic ketoacidosis (EDKA) is a subtype that presents with lower serum glucose in the setting of metabolic acidosis and ketosis. EDKA was first described by Munro in nearly 20% of their series of DKA cases [1]. Possible causes of EDKA in this series include insulin use, decreased calorie intake, pregnancy, pancreatitis, alcohol consumption, cocaine intoxication, chronic liver disease, glycogen storage disorders, prolonged vomiting, and diarrhea [2]. Sodium-glucose cotransporter-2 inhibitors (SGLT2i) were introduced in 2013 as anti-hyperglycemic medications. SGLT2i are gaining traction for diabetic kidney disease and cardiac protection in patients with diabetes and even showing improvements in cardiovascular and kidney disease in patients without diabetes [3-8]. The purported mechanism of action is inhibiting glucose reabsorption in the proximal tubule by 30%-50%, resulting in glucosuria. Importantly, these drugs lower serum glucose through an insulin-independent mechanism. In addition to the mortality benefit and decreases in glucose, these drugs reduce body weight, blood pressure, and albuminuria [9-10]. In 2015, the US Food and Drug Administration (US FDA) announced cases of DKA developed while taking SGLT2i [11]. Blau et al. reviewed the US FDA Adverse Event Reporting System and uncovered that 71% of reported DKA cases with SGLT2i were euglycemic [12-13]. He noted that many of these cases occurred in patients with type 1 diabetes despite the lack of US FDA approval of SGLT2i use in this population [13]. A recurring theme in those early cases of EDKA was a delay in diagnosis and treatment. The lack of awareness of the infrequent adverse effect, in combination with an unremarkable glucose level, lead to delays in diagnosis and treatment [14].

The method by which SGLT2 inhibitors can directly cause EDKA is unclear. Sodium-glucose cotransporter 2 is a transmembrane protein located on the apical side of the proximal convoluted tubules, which reabsorbs filtered sodium and glucose from the tubular fluid [15]. SGLT2 inhibitors (gliflozins) lower blood glucose through renal losses as opposed to other antidiabetic medication mechanisms, including reducing hepatic glucose production, increasing cellular uptake of glucose, or inhibiting carbohydrate absorption in the intestines. This can result in hypoglycemic suppression of insulin release leading to ketosis. Additionally, SGLT2i may directly stimulate glucagon. Low insulin levels stimulate two pathways. First, it induces lipolysis-producing free fatty acids, which undergo beta-oxidation producing acetyl-CoA, which is converted into ketone bodies. Second, low insulin levels stimulate acetyl-CoA carboxylase activity to create malonyl-CoA, an inhibitor of carnitine palmitoyl-transferase (CPT-1). By preventing CPT-1 activity, insulin transport is impaired. Both mechanisms lead to beta-oxidation and the ketosis characteristic of EDKA [16-17]. Ketogenesis produces three ketones: 3-beta-hydroxybutyrate, acetoacetate, and acetone. The anion gap metabolic acidosis in DKA arises from increasing serum levels of beta-hydroxybutyrate and acetoacetate, which are strong acids. Beta-hydroxybutyrate is the dominant ketone found in the serum initially in DKA until acetoacetate accumulates later. Acetone is not an acid and is highly soluble in water. This characteristic of acetone is responsible for the “fruity” smell of breath in some DKA patients [18].

Ketogenic diets also increase blood ketones. A ketogenic diet consists of a very low-carbohydrate and high-fat diet. This diet has gained popularity due to its favorable cardiovascular benefits, including rapid weight loss, decrease in serum hemoglobin A1c, and decrease in total cholesterol [19-20]. This diet has also been shown to be beneficial for patients on polydrug therapy for seizure disorders, slow the progression of Alzheimer’s disease, and improve other neurodegenerative diseases [19]. Although all long-term effects are not well-established, this diet is controversial for its potential adverse effects, including dyslipidemia, glucose homeostasis, and liver steatosis [21]. For those with a diagnosis of diabetes, a low-carbohydrate and ketogenic diet is debated due to the life-threatening complications of ketoacidosis [22]. By following a low carbohydrate diet, blood glucose and insulin levels are reduced, which decreases glycolysis activity producing low oxaloacetate concentrations for metabolism in the citric acid cycle. Low citric acid cycle activity shifts the body’s primary metabolism toward beta-oxidation leading to elevated plasma ketone body production [23]. This shift in primary fuel source occurs over two to three days of fasting [23]. This process is distinct from diabetic ketogenesis because diabetic ketone body clearance is impaired in addition to increased ketone production. While a ketogenic diet provides an alternate source of energy in a healthy person, diabetic ketoacidosis is a repercussion of metabolic dysregulation [24]. Accordingly, starting a ketogenic diet in diabetic patients has been recorded to precipitate ketogenic acidosis [25]. Additionally, there are some cases reporting EDKA in diabetic patients adhering to a ketogenic diet and an SGLT2i. One case even described the successful reimplementation of a ketogenic diet after an EDKA episode in the setting of a low carbohydrate diet and SGLT2i. After cessation of the SGLT2i and close initial monitoring of diet reimplementation, the patient safely remained in ketosis for two years [26].

This report presents a case of euglycemic diabetic ketoacidosis in a 51-year-old woman with type 2 diabetes mellitus on canagliflozin and recent sleeve gastrectomy. This case highlights several important aspects. First, SGLT2 inhibitors must be managed carefully in the perioperative setting. Next, the patient, in this case, presents with hypokalemia before insulin treatment as opposed to the common occurrence of hypokalemia after insulin treatment [27]. Thirdly, a complete reversal of metabolic acidosis took longer than expected at about two weeks after treatment. Lastly, we will examine the claim to contraindicate SGLT2 inhibitors in patients with a history of euglycemic diabetic ketoacidosis.

## Case presentation

Presentation

A 51-year-old female presented one day after laparoscopic-assisted sleeve gastrectomy with lethargy and cold extremities. Her past medical history included type 2 diabetes mellitus, hypertension, hypercholesterolemia, gastroesophageal reflux disease, and morbid obesity. Her diabetes medications included insulin aspart, insulin glargine, metformin, and canagliflozin. To prepare for surgery, canagliflozin, an SGLT2 inhibitor, was discontinued two days prior to surgery. Additionally, the patient reported a diet consisting of three protein shakes per day for three weeks prior to surgery.

Investigation

On examination, she was afebrile, tachycardic with a pulse of 130, hypertensive with a systolic pressure of 200, and had strong pedal pulses bilaterally with cool extremities. Laboratory evaluation revealed modest hyperglycemia with serum levels ranging from 150 to 180 mg/dL and lactic acid of 1 mmol/L. Additionally, the lab revealed low serum bicarbonate of 8 mmol/L with an elevated anion gap of 37 mmol/L. Arterial blood gas showed a pH of 7.21 and partial pressure of carbon dioxide (pCO2) of 60. Urine analysis was positive for ketonuria and glucosuria. Therefore, labs revealed a normoglycemic anion gap metabolic acidosis with ketones in the urine consistent with the diagnosis of EDKA.

Repeat blood work four hours later showed potassium of 2.6 mmol/L and bicarbonate of 4 mmol/L, which were the lowest potassium and bicarbonate values of her hospital stay. On postoperative day two, the patient’s mentation seemed to be altered, as she was groggy but arousable. Blood urea nitrogen and creatinine increased to 22 mg/dL and 1.21 mg/dL, respectively, indicating acute kidney injury (AKI).

Management and outcome

After starting initial fluid resuscitation with lactated ringers, an intravenous (IV) insulin drip as well as 5% dextrose diluted in water, the resuscitation fluid was switched to normal saline. Hypokalemia was treated with oral and IV potassium chloride.

By the third hospital day, hypertension was well-controlled, the anion gap closed, and hypokalemia resolved. Hypercalcemia improved with IV fluids. The patient’s mental status also recovered. Insulin drip was discontinued at this time while insulin detemir was started and solid food consumption was encouraged.

By day four of her hospital stay, the patient was transferred out of the ICU. Home medications were reintroduced including short and long-acting insulin. Metformin and GLP-1 agonists were held until evaluation in the outpatient office for follow-up. SGLT2i was added to the patient’s allergy list with a life-threatening side effect of euglycemic DKA.

Serum bicarbonate was stable above 15 mmol/L since day four, and she was discharged on postoperative day six with a bicarbonate level of 16 mmol/L. On postoperative day 15, blood work revealed a bicarbonate level of 30 mmol/L.

## Discussion

We present a 51-year-old female with type 2 diabetes mellitus on an SGLT2i and a ketogenic diet for three weeks prior to bariatric surgery. Laboratory studies indicated EDKA and subsequent AKI. The patient stopped canagliflozin 150 mg daily two days prior to surgery. Canagliflozin has a half-life between 10.6-13.1 hours. Therefore, between 3.12% and 6.25% remained in the patient’s system at the time of surgery. In March 2020, FDA announced canagliflozin, dapagliflozin, and empagliflozin should be stopped three days prior to scheduled surgery as they are associated with acidosis and serious urinary tract infections [28]. Similarly, an international consensus review on SGLT2 inhibitors mentioned holding the medication three days before a procedure, especially in patients with decreased calorie intake [29]. However, Pace et al. recommended holding SGLT2 inhibitors preoperatively for at least five days because of the relatively long half-life and serious complication of EKDA [30]. It is crucial to be vigilant about the possibility of a prolonged half-life of SGLT2i in patients with decreased renal function. These patients may need to consider holding medications for longer than the recommended amount of time. Aside from the proper holding time of medication, high-risk patients are not well-characterized. Meyer et al. exposed identifiable precipitants in a series of SGLT2i-associated DKA cases, which indicated risk mitigation potential [31]. In the literature review, there are few similar DKA cases available regarding patients taking SGLT2i against the backdrop of a low carbohydrate diet [32-35]. Shaikh et al. and Tauseef et al. both share EDKA examples of diabetics on SGLT2i and ketogenic diets between one to three weeks [32,34]. These cases underscore the importance of considering EDKA as a postoperative complication even after appropriate measures are taken to hold SGLT2 inhibitors. Recurring signs and symptoms to monitor for SGLT2i-associated EDKA complications include altered mental status, polyuria, polydipsia, gastrointestinal disturbances (vomiting, abdominal pain), glucosuria, tachypnea, tachycardia, dehydration, or ketosis [36]. Since we do not have preoperative labs on our patient, reported similar cases support the possibility that our patient could have already been in EDKA prior to surgery. In this case and others, there is potential to avoid the occurrence of DKA through detailed dietary counseling by establishing modifiable risk factors. This case emphasizes the need for further awareness and education of predisposing risk and perioperative management of SGLT2 inhibitors.

Before insulin treatment was started, the patient's blood work revealed hypokalemia. Arora et al. estimated a 5.6% prevalence of hypokalemia in patients with DKA [37]. The cause of hypokalemia in DKA can be explained by osmotic diuresis. Glucosuria induces volume loss, which prompts the release of aldosterone and, subsequently, potassium secretion [27]. Additionally, ketones are not reabsorbable anions in the distal tubule leading to increased urinary potassium loss. Therefore, total body potassium is generally low in DKA and can reveal itself as hypokalemia. However, hyperkalemia is a more common finding in DKA. This is explained by insulin’s role in the activation of sodium-potassium ATPases, responsible for the movement of potassium into cells. In the insulin-depleted environment of DKA, potassium remains in the extracellular compartment and may manifest as hyperkalemia. High serum potassium can predispose patients to fatal cardiac arrhythmias [37]. However, in patients that present with hypokalemia, treating DKA with insulin can result in profound, symptomatic hypokalemia (<2.5) affecting neuromuscular and cardiopulmonary systems. Severe symptoms can range from muscular necrosis, ascending paralysis, cardiac arrhythmias, respiratory arrest, etc. [12,19-20,38]. Therefore, the American Diabetes Association guidelines on DKA recommend checking and correcting any hypokalemia before starting IV insulin.

The therapeutic intervention of DKA targets volume restoration, clearing blood ketones, and correcting any electrolyte imbalance. Our patient’s anion gap closed on day three, leaving a non-anion gap metabolic acidosis that persisted for up to 15 days postop. Freire et al. define prolonged ICU and hospital length of stay as three or more and six or more days, respectively [39]. A review of DKA hospitalizations in 2018 revealed a decreasing average length of stay between 2003 and 2014 with the average being 3.24 days in 2014 [40]. Accordingly, the duration of metabolic acidosis reversal caused a prolonged hospital stay in this patient. Although current management guidelines do not describe extended acidosis periods in EDKA, there are SGLT2 inhibitor-associated EDKA cases reported taking longer to treat than DKA in non-users of the medication [29,41-43]. A Korean study revealed a significant ICU stay for DKA lasting four days in SGLT2i users as opposed to the two days of non-users [41]. However, there have been few cases describing long treatment times for EDKA in particular. In a Japanese case report, one dose of an SGLT2 inhibitor in a patient with a low-calorie diet caused EDKA lasting about 60 hours after treatment was started [42]. In Ireland, two postoperative SGLT2 inhibitor-associated EDKA patients took 92 hours to fully recover from ketoacidosis after initiation of treatment [43].

In this patient’s allergy list, SGLT2 inhibitors are recorded with a life-threatening reaction of EDKA. The US FDA directs patients to stop taking SGLT2 inhibitors if they experience symptoms of ketoacidosis or if medical providers suspected ketoacidosis as treatment should be implemented promptly [28]. However, recommendations on reinstituting SGLT2 inhibitors after an episode of EKDA on the medication is unclear. Very few of the available SGLT2i-associated EDKA cases disclose their follow-up management describing if the medication was discontinued or reinitiated after EDKA recovery. One case of EDKA in the setting of SGLT2i and hypertriglyceridemia suggested consulting with an endocrinologist before restarting an SGLT2i [44]. Another recommended close follow-up with nephrology only [36]. In this case, there may be multiple precipitating factors leading to the development of EDKA including the use of an SGLT2i, ketogenic diet, and the physiologic stress of surgery. It is uncertain if the patient would have experienced EKDA if she did not follow a ketogenic diet or undergo surgery. These intricate risk factors and inciting events require more study to investigate the possible reinitiation of SGLT2 inhibitors in patients where the drug has been shown to prolong life.

## Conclusions

As SGLT2i are increasingly prescribed, the reports of associated adverse effects like EDKA are also rising. EKDA is characterized by severe metabolic acidosis, ketosis, and serum glucose within normal limits. Our case illustrates SGLT2i-associated EDKA in the setting of ketosis-inciting factors including a ketogenic diet and undergoing the physiologic stress of an operation. Low-carbohydrate intake is known to produce ketones as a primary source of energy and, ultimately, can contribute to ketoacidosis in a patient prone to metabolic dysfunction such as diabetes. Therefore, it is important for clinicians to review modifiable risk factors like diets in patients taking SGLT2i. Moreover, this EDKA case was atypical in the presentation of hypokalemia instead of the more common measurement of hyperkalemia. Vigilant management was highlighted by testing and correcting hypokalemia and avoiding further worsening of hypokalemia with IV insulin before electrolyte correction. The next steps for future study would evaluate the safety of reintroducing an SGLT2 inhibitor after an episode of euglycemic diabetic ketoacidosis.
